# The efficacy and safety of *Codonopsis lanceolata* water extract for sarcopenia: A study protocol for randomized, double-blind, placebo-controlled clinical trial

**DOI:** 10.1097/MD.0000000000030773

**Published:** 2022-09-30

**Authors:** Jaehyeon Park, Hyungsuk Kim, Se-Young Choung, Yong Jae Jeon, Hyo Jin Jeon, Sang Jun Youn, Yong Choi, Hong-Seok Cho, Junhyuk Kang, Yeonho Seo, Koh-Woon Kim, Mi-Yeon Song

**Affiliations:** a Department of Korean Rehabilitation Medicine, College of Korean Medicine, Kyung Hee University, Seoul, Republic of Korea; b Department of Preventive Pharmacy, College of Pharmacy, Dankook University; c MILAE Bioresources Co., Ltd., Seoul, Republic of Korea; d RnBS Corp.

**Keywords:** *Codonopsis lanceolata*, protocol, randomized controlled trial, sarcopenia

## Abstract

This study aimed to propose a protocol to demonstrate the efficacy of *Codonopsis lanceolata* water extract for the improvement of skeletal muscle mass (SMM) and function (muscle strength or performance function) and its safety compared to a placebo in adults with reduced muscle strength.

**Methods and analysis:**

A randomized double-blind placebo-controlled clinical trial was conducted. Participants will be recruited from the Korean Medicine Hospital in South Korea. One hundred and four adults with reduced muscle strength will be randomly assigned a 1:1 ratio to either the experimental or placebo comparator groups. The participants will consume the product corresponding to their assigned group for the following 12 weeks, and efficacy and safety tests will be conducted. This is the first clinical trial of *C lanceolata* water extract in adults with reduced muscle strength. The results of this study would provide a clinical basis for the efficacy and safety of *C lanceolata* water extract in patients with sarcopenia.

**Ethics and dissemination:**

This trial was approved by the Institutional Review Board (IRB) of Kyung Hee University Korean Medicine Hospital at Gangdong on July 15, 2021 (amendment number: MLB_DDE_H01 [ver. 01]). When a change was made in the clinical trial plan, the IRB reviewed and approved the revised clinical trial plan. The study was registered on the Clinical Research Information Service website on December 3, 2021 (registration number: PRE20211203-003; https://cris.nih.go.kr/cris/search/detailSearch.do?seq=20841&status=1&seq_group=20841&search_page=M). The results of this clinical trial will be reported in the future. Every document related to the clinical trial, such as the electronic case report form, will be recorded and classified by the subject identification code and not by the subject name.

**Strengths and limitations of this study**

- This study is the first to explore the efficacy of a *Codonopsis lanceolata* water extract in adults with reduced muscle strength.
- This was a double-blind, placebo-controlled, randomized trial that minimizes possible bias.
- For the outcomes, we considered various factors to evaluate the muscle strength of the participants: SMM; muscle strength; and physical performance.

## 1. Introduction

Sarcopenia refers to a decrease in skeletal muscle mass (SMM) distributed in the extremities,^[1]^ including loss of SMM and strength, and decreased physical performance.^[1]^ Physical activity tends to be reduced because of limited exercise and excessive sitting.^[2]^ Muscle mass begins to decline after the age of 30 years, and shows a marked decrease with increasing age.^[3]^ The prevalence of sarcopenia is approximately 6% to 15% in patients aged ≥ 65 years^[4]^ and increases to >50% in individuals over the age of 80.^[5]^ The mechanisms of sarcopenia include increased fat accumulation in the skeletal muscle, dysregulation of proteasomal pathways, mitochondrial dysfunction, decreased number of satellite cells, increased reactive oxygen species, and inflammation.^[6]^

The 2010 European Working Group on Sarcopenia in Older People (EWGSOP)^[2]^ added “muscle function” to the existing definition of sarcopenia, which only contained “low muscle mass.” The 2019 revised diagnostic criteria proposed by the EWGSOP^[8]^ suggest low muscle strength, low muscle quantity or quality, and low physical performance. The 2014 Asian Working Group for Sarcopenia (AWGS)^[9]^ definition is similar, except for the cutoff value for Asians due to differences in ethnicity, body size, or cultural backgrounds. In 2019, the AWGS^[10]^ maintained the previous definition, but there were modifications to the diagnostic algorithm and protocols. As the concept of sarcopenia is new, its definition and cutoffs^[11,12]^ have been discussed and revised to achieve consensus.

Sarcopenia increases the incidence of congestive heart failure and severe activities of daily living (ADL) disorders by 1.3 times.^[13]^ Sarcopenia can also increase stroke incidence by 1.4 times^[13]^ and mortality rates by 2.2 times.^[14]^ More than 50 million adults worldwide suffered from sarcopenia in 2000, and more than 200 million patients are expected to develop sarcopenia after 2040.^[2]^ Hospitalization costs reached $40.4 billion in the United States in 2014.^[15]^ Therefore, sarcopenia is a complicated issue that causes physical disability, lowers independent ADL and quality of life, and increases medical expenses.

Regular physical activity and a nutritious diet with protein-calorie hyperalimentation are the most important factors to reduce sarcopenia.^[16]^ Treatment includes exercise, commercial nutraceuticals, drugs, and whole-body electromyostimulation. Drugs and whole-body electromyostimulation have been adopted for treatment in clinical trials, whereas exercise and administration of nutraceuticals have been adopted for more than 70% of treatments.^[17]^ However, to the best of our knowledge, there are no FDA-approved treatments or drugs that have been developed for sarcopenia.

*Codonopsis lanceolata* is native to traditional medicine in East Asian countries, such as Korea and China, because of its tonic, antipyretic, calming cough, expectoration, detoxification, and drainage properties. Non-clinical studies have reported that *C lanceolata* is effective in regulating fat metabolism and suppressing obesity.^[18]^ It may also suppress the onset of obesity and hyperlipidemia by inhibiting weight gain and body fat accumulation and reducing serum triglyceride and total cholesterol levels.^[19]^ *C lanceolata* could improve skeletal muscle atrophy by increasing muscle protein synthesis, by activating protein kinase B, phosphatidylinositol-3-kinase, and the mammalian target of rapamycin complex 1 pathway. It may reduce muscle protein degradation by inhibiting the expression of muscle atrophy F-box protein (Atrogin-1) and muscle ring finger-1 (MuRF1),^[20]^ and is also effective in sarcopenic obesity by inhibiting the accumulation of lipids in the skeletal muscle by restoring the activating protein kinase B and phosphatidylinositol-3-kinase pathways and lipid metabolism.^[21]^ However, few clinical trials have directly used *C lanceolata* water extract for the treatment of sarcopenia.

This clinical trial utilized a large-scale randomized controlled trial to evaluate the efficacy of *C lanceolata* water extract for the improvement of SMM and function compared to a placebo for adults with reduced muscle strength.

## 2. Methods

This randomized, double-blind, parallel, placebo-controlled, superiority clinical trial was conducted and designed according to the Standard Protocol Items: Recommendations for Interventional Trials (SPIRIT) 2013 statement. Participants were recruited at the Kyung Hee University Korean Medicine Hospital at Gangdong, Seoul, Korea. The researcher explained the purpose and contents of the clinical trial to the subjects and obtained their written consent.

Subjects who have agreed to participate in the clinical trial will be given a screening number, and the screening test will be conducted within 14 days of their baseline visit. If the screening test results meet both the inclusion and exclusion criteria, the participants will be randomly assigned 1:1 to the experimental or placebo comparator group. Participants will consume the product corresponding to the assigned group for the next 12 weeks. After the baseline visit, 2 visits will be made at the 6th and 12th weeks, and efficacy and safety tests conducted and evaluated (Table 1).

**Table 1:** Clinical trial schedule.

Assessment		Screening	Ingestion period			Out of schedule visit
		D-14~D-1	D0 (0wk, Baseline)	D42 ± 7 (6wk)	D84 ± 7 (12wk)	
Subject consent		○				
Screening numbering		○				
Demographics questionnaire		○				
Body measurement		○	○	○	○	(○)
Lifestyle questionnaire		○			○	(○)
Vital signs		○	○	○	○	○
Medical history and comorbidity		○	○			
Physical test		○	○	○	○	○
Medication investigation		○	○	○	○	○
Laboratory test		○			○	(○)
Pregnancy test (for women of childbearing potential)		○			○	(○)
Subject suitability assessment		○				
Randomization			○			
IPAQ			○		○	(○)
Dietary Questionnaire (24 h recall)			○		○	(○)
Functional test	DXA		○		○	(○)
	Muscle strength (Hand grip)	○	○	○	○	(○)
	SPPB		○	○	○	(○)
	TNF-α, IL-6, IGF-1		○		○	(○)
Product prescription			○	○		
Adverse event				○	○	○
Adherence assessment				○	○	(○)

DXA = dual-energy X-ray absorptiometry, IGF-1 = insulin-like growth factor-1, IL-6 = interleukin-6, IPAQ = International Physical Activity Questionnaire, SPPB = short physical performance battery, TNF-α = tumor necrosis factor-α.

### 2.1. Ethics and dissemination

This trial was approved by the Institutional Review Board (IRB) of Kyung Hee University Korean Medicine Hospital at Gangdong on July 15, 2021 (Number: MLB_DDE_H01 [ver. 01]). When a change was made in the clinical trial plan, the IRB reviewed and approved the revised plan. The study was registered on the Clinical Research Information Service website on December 3, 2021 (registration number: PRE20211203-003; https://cris.nih.go.kr/cris/search/detailSearch.do?seq=20841&status=1&seq_group=20841&search_page=M). The results of this clinical trial will be reported in the future. Every document related to the clinical trial, such as the electronic case report form (eCRF), will be recorded and classified by the subject identification code and not by the subject name.

### 2.2. Recruitment strategy

This study was conducted using bus advertisements and posters. Participants were recruited until June 2022. The clinical trial will be conducted for 12 weeks, and participants will receive medical examinations and consultations during the study period. Their expenses for examinations, consultations, and products for clinical trials will be waived, and their transportation fees for each visit reimbursed. The external monitor will evaluate the recruitment process and suitability of the selection after screening on a monthly basis through regular visits and/or phone calls.

### 2.3. Participants

#### 2.3.1. Inclusion criteria

The researcher enrolled at least 104 adult participants with reduced muscle strength. We included both males and females aged 50 to 85, with reduced muscle strength in a hand grip test measured with a TKK-5401 digital grip dynamometer (Takei Scientific Instruments Co. Ltd., Tokyo, Japan) as follows: aged 50 to 65 years, <30 kg (men) and <20 kg (women); 65 to 85 years, <26 kg (men), and <18 kg (women).

#### 2.3.2. Exclusion criteria

Patients with serious lower extremity musculoskeletal abnormalities or injuries were excluded. Similarly, we excluded patients: with kidney disease requiring treatment or serum creatinine > 2.0mg/dl; liver disease requiring treatment or whose AST or ALT level was ≥ 3 times the upper limit of normal; uncontrolled diabetes mellitus (HbA1c > 7.0%); uncontrolled hypertension; psychologically significant medical history or receiving medication for psychiatric disorders; a history of malignancy within 5 years or receiving treatment (except skin tumors other than melanoma); the inability to talk or walk due to Alzheimer’s disease, vascular dementia, or Parkinson’s disease; fragile hip fractures; myocardial infarction history, coronary artery bypass graft, percutaneous coronary intervention or active unstable angina, heart failure classified as Class III/IV by the New York Heart Association, severe heart valve disease or defect, stroke, and untreated atrial fibrillation, within 3 months; history of drug abuse or drug addiction, and subjects who had taken oral steroids or adrenocorticoids within the last 4 weeks; regular alcohol consumption; an allergic reaction to the investigational product (IP); recent use of dietary supplements related to muscle strength improvement within 2 weeks; continuous participation in aerobic or resistance exercises, or similar clinical trials, within the last 3 months; unsuitable by the researcher for other reasons. Patients taking intermittent medications for sleep disorders or appropriately treated depression were included in the test.

### 2.4. Medical history and medications

During screening, medical history and comorbidities were obtained using questionnaires and medical records. During the baseline visit, the subjects answered whether any disease had developed after screening.

The researcher will review prior and concomitant medication use and record prior medication taken by the subject within 4 weeks before the clinical trial. In addition, the researcher will record concomitant medications that will be used during the clinical trial. If the subject takes drugs that may affect the results, they will be able to register for this clinical trial after 2 or more weeks of a wash-out period. The researcher will also check for concomitant medications at 6 and 12 weeks after the baseline visit.

### 2.5. Randomization and allocation concealment

Randomization and allocation will be performed by blind stratified block randomization based on the subjects’ sex at the baseline visit. Each subject was randomly assigned in a 1:1 ratio to either the test or placebo product. Randomization uses an interactive web response system. The randomization code enables reproduction through seed allocation. Each subject was assigned a unique IP number via the interactive web response system. Each subject was provided with a corresponding test or placebo product, according to their IP number.

The blinded sponsor, who was uninterested in this clinical trial, strictly managed and kept both the randomization codes and IP numbers closed until the statistical analysis.

### 2.6. Blinding

To maintain anonymity, every packaging material will be identical, except for the individual IP numbers. Identical packaging materials helped the subjects, researchers, test product managers (or management pharmacists), research nurses, and monitors to be blinded. When information on the intake group affects the treatment of subjects in the case of an emergency, the principal investigator will request unblinding via electronic data capture. Upon request, relevant information was provided according to the standard operating procedure. If a patient requires immediate medical attention, unblinding can be performed immediately. In addition, the researcher notified the sponsor within 24 hours.

### 2.7. Interventions

The test and placebo products, manufactured by the sponsor, were provided to the researcher. Each subject received the products at the baseline and sixth week visits, and the remaining products were recalled at subsequent visits. The test product was a 750 mg white-coated tablet, and the main component was *Codonopsis lanceolate* water extract. The placebo was a 750 mg white-coated tablet, and the main component was crystalline cellulose. Subjects will be instructed to take 1 tablet once a day, with plenty of water before breakfast, for 12 weeks (84 days). The intake status was recorded. In addition, the amount of remaining products will be checked at subsequent visits. The adherence assessment was calculated when adherence was confirmed.

### 2.8. Concomitant medication

At the discretion of the researcher, if the subject had been taking a drug prior to participation in the trial, the subject was allowed to continue taking the drug as long as it was not expected to affect the interpretation of the results of this clinical trial. Subjects may also be allowed to take a concomitant drug when it is temporarily used for the treatment of other diseases or adverse events. Subjects will not be permitted to participate in the study if they consume any drugs or food that may affect muscle mass and muscle strength improvement, which in turn may directly or indirectly affect the results of this study.

### 2.9. Outcomes

#### 2.9.1. Primary outcome

##### 2.9.1.1. Amount of change in short physical performance battery (SPPB) score

The SPPB score was assessed twice: at baseline and at the 12th week visit. The SPPB test, a comprehensive measure of physical performance ability, was used for both research and clinical evaluation of functionality.^[2]^ The SPPB consists of 3 subcomponents: standing balance, gait velocity, and the repeated chair stand test. The gait velocity test was performed twice, and the results are presented as the fastest of the 2 trials. The scoring system for the SPPB is presented as follows: 0, inability to perform; 1 to 4 designated depending on performance. Twelve points were the maximum.

#### 2.9.2. Secondary outcomes

##### 2.9.2.1. Amount of change in hand grip strength

The participant’s hand grip strength was measured at screening, at baseline, and at the subsequent visits using the dynamometer, measuring from 5.0 to 100 kg in 0.1 kg increments. First, the dominant hand was assessed. The hand grip test was performed 3 times, and the results are presented as the highest of the 3 trials.

##### 2.9.2.2. Change in SMM and skeletal muscle index (SMI)

**Figure 1.**
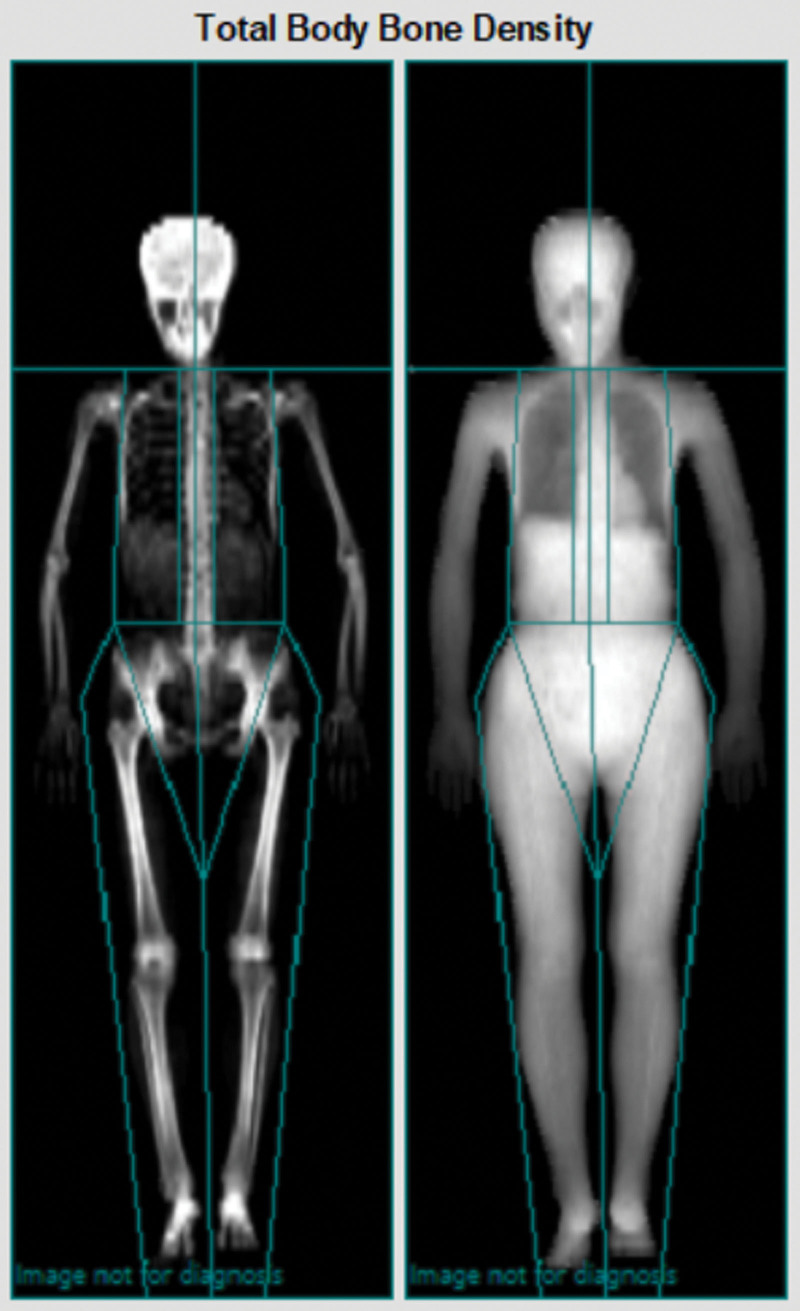
Measuring method of appendicular skeletal muscle mass by dual-energy X-ray absorptiometry.

SMM was measured using dual-energy X-ray absorptiometry (DXA) at the baseline and 12th week visits. The subject removed all wearable items, except light clothing, and the examination proceeded by continuous transverse imaging from head to toe. SMI was calculated using the following formula: SMI = SMM (kg)/body weight (kg) × 100 at the baseline and 12th week visits. Changes in appendicular skeletal muscle mass (ASM) were measured using DXA at the baseline and 12th week visits, which is the sum of lean soft tissue from both the upper and lower extremities (Fig. 1). Change in ASM index (ASMI) was calculated using: ASMI = ASM (kg)/height2 (m2) at baseline and 12th week visits.

##### 2.9.2.3. Amount of change in body fat

Body fat mass (BFM), percentage of body fat (%BF), and free fat mass (FFM) were measured using DXA. BFM and %BF were measured using DXA at baseline and 12th week visits. FFM was calculated using the following formula: FFM = LEAN (muscle) + bone mineral content at baseline and at the 12th week visit.

##### 2.9.2.4. Amount of change in SPPB score

SPPB score was assessed at the baseline and sixth week visits. The change in score for each SPPB item (standing balance test, gait velocity test, and repeated chair stand test) were assessed at the baseline and at subsequent visits.

##### 2.9.2.5. Amount of change in tumor necrosis factor-α (Tnf-α), interleukin-6 (IL-6), and insulin-like growth factor-1 (IGF-1)

TNF-α, IL-6, and IGF-1 levels were measured at baseline and 12th week visits.

##### 2.9.2.6. Adverse events

The researcher will evaluate adverse events at the sixth and 12th week visits through non-directive questioning of participants. The participants may have voluntarily reported any adverse events. Physical tests, laboratory tests, and other assessments can be performed to identify adverse events.

##### 2.9.2.7. Laboratory tests and vital signs

Hematologic examination, blood chemistry examination, and urinalysis tests are performed at screening and at the 12th week of visits. Systolic blood pressure, diastolic blood pressure, pulse, and body temperature were measured at the screening, baseline, and both subsequent visits. Blood pressure and pulse were measured using the same FT-500R PLUS automatic blood pressure monitor (Jawon Medical Co. Ltd., Gyeongsan, Republic of Korea) after a 10 minutes rest.

### 2.10. Sample size

To calculate the number of subjects, the change in SPPB score, which measures muscle functionality, was used. The type I error rate (α) was 0.05, and the type II error rate (β) was 0.15; thus, the power of the test (1-β) was 85%. The effect size of the difference in the amount of change in SPPB score between the experimental and placebo comparator groups was assumed to be 0.6667. Due to the lack of previous clinical trials, a meaningful change in SPPB score to calculate the number of subjects was assumed to be 1.0.^[22]^ The coefficient of variance for the difference in the amount of change in SPPB score was assumed to be 1.5. Based on this information, 41 subjects were included in each group. Considering a dropout rate of 20%, 52 subjects in each group. Therefore, 104 subjects were required.

### 2.11. Statistical analysis

The main analysis group for efficacy evaluation used the per-protocol set (PPS), which consisted of protocol-compliant subjects who completed the clinical trial. The secondary analysis group used the full analysis set based on the intention to treat that all subjects who have been randomly assigned should be the targets of the evaluation. Safety evaluation was performed on the safety analysis set, which included subjects whose safety information was collected more than once.

The significance level of the statistical analysis was set at 5% and a 2-tailed test was used. Continuous variables represent the number of participants, mean, and standard deviation. Categorical variables were presented as frequencies and percentages. The subjects’ demographic information, medication, and medical history presented the results of the PPS analysis group. To address missing full analysis set values, the last observation carried forward analysis was applied. To deal with the missing values of PPS and safety analysis set, the observed case approach, which analyzes missing values without correction, was used.

The mean, standard deviation, median, minimum value, and maximum value for the amount of change in the SPPB score, score for each SPPB item, hand grip, SMM, SMI, ASM, ASMI, BFM, %BF, FFM, TNF-α, IL-6, and IGF-1 are presented for each group. The least square mean (LSmean) and standard error of the amount of change corrected for the independent variable of the fitted general linear model are presented for each group. Comparisons between groups was analyzed by fitting a general linear model. If any factor affecting the outcome variable is identified, the general linear model is fitted by adding the factor as an independent variable. For data bias, the Wilcoxon rank-sum test, which is a non-parametric method, was applied. In addition, an analysis based on stratification factors was performed. Changes in the experimental or placebo comparator groups will be analyzed using a paired t-test or Wilcoxon signed-rank test.

The number of subjects with adverse events, drug adverse events, serious adverse events, incidence rate, 95% 2-tailed confidence interval, and the number of occurrences will be presented for each group. Differences in incidence rates between groups are analyzed using the chi-square test or Fisher’s exact test.

In terms of laboratory tests, continuous data were presented as the mean and standard deviation for each group. Differences in the amount of change between the groups were analyzed using an unpaired *t* test (or Wilcoxon’s rank-sum test). Pre- and post-ingestion comparisons were analyzed using a paired *t* test (or Wilcoxon’s signed-rank test). Categorical data were presented as a shift table in terms of frequency and percentage. In addition, the comparison between pre-and post-ingestion will be analyzed using McNemar’s test.

In terms of vital signs, mean and standard deviation are presented for each group. The mean difference in the amount of change between groups was analyzed using an unpaired *t* test (or Wilcoxon’s rank-sum test). Pre- and post-ingestion comparisons were analyzed using a paired *t* test (or Wilcoxon’s signed-rank test).

### 2.12. Adverse events

Subjects will be assessed for any adverse events during their visits and/or investigation. If a serious adverse event occurs, appropriate tests and follow-ups should be performed to prevent unnecessary risk. In addition, the principal investigator or researcher should notify the monitor and report to the IRB on the SOP of the institution. All adverse events must be reported for eCRF. If any necessary risk occurs to the subject, compensation should be made in accordance with the rules for compensation for damage.

The severity of adverse events was evaluated as mild, moderate, or severe, based on the maximum intensity. Causal relationships with products for clinical trials were classified into 6 categories according to the World Health Organization Uppsala Monitoring Center criteria.

### 2.13. Stopping rules

Subjects may withdraw consent at any time for any reason without a disadvantage. In addition, a subject may withdraw for any reason. Subjects who discontinued the trial will complete the efficacy and safety assessments, with the exception of declining revisits.

Subjects may be withdrawn by the researcher if: a violation of the inclusion/exclusion criteria is found during the clinical trial; the clinical trial is discontinued due to a sudden accident; their safety might be compromised for any reason; a serious adverse event including excessive predicted symptoms or death occurs; they take contraindicated drugs or the researcher assesses whether the subject requires any treatment with contraindicated drugs; the researcher assessed that they were no longer able to participate in the study due to inappropriate dosing of IP; the subject is lost to follow up; they suffer a worsening of symptoms; and if researcher assesses they are no longer able to participate due to other reasons. The subject may withdraw from or discontinue the study if they or a legal representative withdraws consent, if they suffer an adverse event, or any other reason, such as their refusal to consume the IP or non-cooperation.

This clinical trial may be prematurely terminated at the subject’s request, the occurrence of an adverse event, serious protocol violation, or other plausible reasons. The principal investigator has the final authority to discontinue or modify a clinical trial. In this case, the principal investigator promptly notifies the monitor by phone or by writing.

### 2.14. Data management and monitoring

Third-party researchers are responsible to enter subject data collected for clinical trials. The subject data were recorded using the assigned IP numbers for blinding. The data file is protected with a password, and a third-party researcher has the right to access it. Subjects’ prior and concomitant medications will be coded using the Anatomical Therapeutic Chemical code, and medical history and adverse events will be coded using the World Health Organization Adverse Reaction Terminology or Medical Dictionary for Regulatory Activities. Documents related to clinical trials must be retained for 3 years after the end of the clinical trial.

Clinical trial monitoring activities should be conducted using an external monitor via phone or regular visits to the trial site. The external monitor checks whether the supporting documents and the eCRF match, and monitors whether the clinical trial is performed according to the plan and related guidelines. Monitoring based on the relevant SOP ensures that the data collection, records, documents, and reports comply with the Korean Good Clinical Practice and related regulations.

### 2.15. Patient and public involvement

None.

## 3. Discussion

EWGSOP defined grip strengths of <30 kg for men and 20 kg for women as cutoffs in the diagnostic criteria for sarcopenia, and AWGS defined grip strengths of <26 kg for men and 18 kg for women. We considered these criteria with respect to age when developing our inclusion criteria. For those aged 65 to 85 years, grip strengths of <26 kg for men and <18 kg for women were included.^[2,9]^

The incidence of sarcopenia has gradually increased due to a lack of physical activity and aging of the population. Unfortunately, sarcopenia may cause diseases, such as congestive heart failure or stroke. Additionally, it can lower quality of life. To treat sarcopenia, it is important to develop drugs for direct improvement, other than treatments such as regular exercise and a healthy diet, which are recommended in clinical practice.^[16]^

*C lanceolata* has shown positive changes in muscle.^[20,21]^ Therefore, this high-quality randomized controlled trial protocol was designed for a large-scale, double-blind clinical trial using *C lanceolata* water extract. This study is the first clinical trial of *C lanceolata* water extract in adults with reduced muscle strength. The results of this study would provide a clinical basis for the efficacy and safety of *C lanceolata* water extract in patients with sarcopenia, and this evidence will be useful to patients, doctors, and researchers. Through this study, we expect to see a reduction in diseases and disabilities caused by sarcopenia, and an improvement in ADL and quality of life.

## Author contributions

KWK and HSC developed the study design and study protocol. SYC conducted non-clinical study. MYS implemented funding acquisition. SJY and YC participated in clinical trial design and clinical trial operation support. YJJ and HJJ contributed to clinical trial design and clinical trial support. JP registered the protocol on the website. JP and HK wrote the original draft and MYS reviewed and edited it. JK and YS will conduct study procedures. MYS is the principal investigator. All authors reviewed the study protocol and approved the final manuscript.

**Conceptualization:** Jaehyeon Park, Hyungsuk Kim, Hyo Jin Jeon, Sang Jun Youn, Yong Choi, Hong-Seok Cho, Mi-Yeon Song.

**Data curation:** Jaehyeon Park, Hyo Jin Jeon, Sang Jun Youn, Yong Choi, Hong-Seok Cho.

**Formal analysis:** Hyungsuk Kim, Hyo Jin Jeon, Sang Jun Youn, Yong Choi, Hong-Seok Cho, Junhyuk Kang.

**Funding acquisition:** Mi-Yeon Song.

**Investigation:** Hyungsuk Kim, Se-Young Choung, Yong Jae Jeon, Hyo Jin Jeon, Sang Jun Youn, Yong Choi, Hong-Seok Cho, Yeonho Seo.

**Methodology:** Se-Young Choung, Yong Jae Jeon, Yong Choi, Hong-Seok Cho, Yeonho Seo, Koh-Woon Kim.

**Project administration:** Se-Young Choung, Yong Jae Jeon, Junhyuk Kang, Yeonho Seo, Koh-Woon Kim.

**Resources:** Se-Young Choung, Hyo Jin Jeon, Junhyuk Kang, Koh-Woon Kim.

**Software:** Jaehyeon Park, Hyungsuk Kim, Se-Young Choung, Yong Jae Jeon, Junhyuk Kang, Koh-Woon Kim.

**Supervision:** Junhyuk Kang.

**Validation:** Yong Jae Jeon, Yeonho Seo.

**Visualization:** Yong Jae Jeon, Yeonho Seo.

**Writing – original draft:** Jaehyeon Park, Hyungsuk Kim, Mi-Yeon Song.

**Writing – review & editing:** Jaehyeon Park, Mi-Yeon Song.
